# Case Report: Novel Biallelic Variants in *DNAJC21* Causing an Inherited Bone Marrow Failure Spectrum Phenotype: An Odyssey to Diagnosis

**DOI:** 10.3389/fgene.2022.870233

**Published:** 2022-04-08

**Authors:** Adela Chirita-Emandi, Carmen-Angela-Maria Petrescu, Cristian G. Zimbru, Florina Stoica, Catalin Marian, Andreea Ciubotaru, Mihaela Bataneant, Maria Puiu

**Affiliations:** ^1^ Department of Genetics, Center of Genomic Medicine, “Victor Babes” University of Medicine and Pharmacy, Timisoara, Romania; ^2^ Regional Center of Medical Genetics Timis, Clinical Emergency Hospital for Children “Louis Turcanu”, Timisoara, Romania; ^3^ Department of Pediatrics, “Victor Babes” University of Medicine and Pharmacy, Timisoara, Romania; ^4^ Department of Oncology and Hematology, Clinical Emergency Hospital for Children “Louis Turcanu”, Timisoara, Romania; ^5^ Department of Automation and Applied Informatics, Politehnica University of Timisoara, Timisoara, Romania; ^6^ Department of Ophthalmology, Municipal Clinical Emergency Hospital of Timisoara, Timisoara, Romania; ^7^ Department of Biochemistry, “Victor Babes” University of Medicine and Pharmacy, Timisoara, Romania; ^8^ Center for Complex Networks Science, “Victor Babes” University of Medicine and Pharmacy, Timisoara, Romania; ^9^ INFOSAN Ophthalmology Hospital, Bucharest, Romania

**Keywords:** DNAJC21 gene, ribosomopathy, bone marrow failure syndrome, Shwachman–Diamond syndrome, telomeres

## Abstract

Bone marrow failure represents an umbrella diagnosis for several life-threatening disorders. In many people, the etiology remains unknown for a long time, leading to an odyssey to diagnosis, with numerous tests performed and sometimes inappropriate treatment. Biallelic pathogenic variants in the *DNAJC21* gene were recently discovered to cause bone marrow failure syndrome type 3, having phenotypic overlap with Fanconi anemia, dyskeratosis congenita, Shwachman–Diamond syndrome, and Diamond–Blackfan anemia. Herein, we report an 8-year-old boy, with normal intellect, presenting bone marrow failure; growth retardation; failure to thrive; recurrent infections (including sepsis); cryptorchidia; skeletal, skin, teeth, and hair abnormalities; joint hypermobility; eczema; palpebral ptosis; high myopia; rod–cone retinal dystrophy; and short telomeres. He underwent several tests and evaluations, including genetic investigations (panel and exome sequencing), before the *DNAJC21* gene was known to cause disease. Whole-genome sequencing performed at the age of 7 years, identified two novel, pathogenic, and compound heterozygous variants in the *DNAJC21* gene: NM_001012339.3:c.148C>T (stopgain-maternal origin), p.Gln50^∗^ and c.643_644delinsTTT (frameshift paternal origin), and p.Lys215Phefs^∗^71. He received aggressive treatments for his multisystem disease: blood cell transfusions, high-dose corticosteroids, immunoglobulins, multiple antibiotics, vitamins, growth hormone, and others. However, allogeneic hematopoietic stem cell transplantation was avoided. The clinical evolution of bone marrow failure and recurrent infections stabilized with age, yet the myopia progressed. Exocrine pancreatic insufficiency was not detected. This report widens the molecular and clinical understanding of bone marrow failure syndrome type 3. Genome sequencing directed a precise diagnosis that improved patient management and enabled family genetic counseling.

## Introduction

Bone marrow failure represents an umbrella diagnosis of several life-threatening disorders. In many people, etiology remains unknown for a long time, leading to an odyssey to diagnosis and numerous tests performed (Bluteau et al., 2018). Biallelic pathogenic variants in the *DNAJC21* gene are recently understood as a cause for bone marrow failure syndrome type 3 (MIM: 617052) (Tummala et al., 2016). The phenotype is overlapping with Shwachman–Diamond syndrome (MIM: 260400), Fanconi anemia (MIM:227650), Diamond–Blackfan anemia (MIM: 105650), and dyskeratosis congenita (MIM:305000) (D’Amours et al., 2018).

At the time of writing this report, 17 people with biallelic *DNAJC21* pathogenic variants were reported. D’Amours et al. reported 15 patients in 2018, while afterward, two more people were reported (Ji et al., 2019; Barhoom et al., 2021). Cardinal features involve multiple organs and systems as follows: bone marrow failure, growth retardation, failure to thrive, developmental delay, recurrent infections, and skeletal, skin, dental, and hair anomalies. Additional features include retinal dystrophy, myopia, astigmatism, pancreatic insufficiency, liver cirrhosis, congenital hip dysplasia, joint hypermobility, cryptorchidism, and short telomeres (D’Amours et al., 2018). People with biallelic *DNAJC21* variants were evaluated differently. The first reports neither evaluated the exocrine function of the pancreas nor presented a detailed eye phenotype (Tummala et al., 2016). Furthermore, even though this group of disorders predispose to myeloid malignancies (Godley, 2017), only two out of 17 patients were reported to have acute myeloid leukemia (one person received a bone marrow transplant). Notably, the people reported had a limited follow-up, the longest being 14 y (D’Amours et al., 2018; Ji et al., 2019). Cytopenia was reported to improve and stabilize with age in some individuals without a bone marrow transplant (Dhanraj et al., 2017, 21; D’Amours et al., 2018). The phenotype, prognosis, and the molecular spectrum of causative biallelic *DNAJC21* variants are insufficiently understood, leading to difficulties in patient management.

To broaden the molecular and clinical understanding of bone marrow failure syndrome type 3, we report the phenotype of a schoolboy, with two novel, pathogenic, compound heterozygous variants in the *DNAJC21* gene, identified using genome sequencing.

## Case Description

**FIGURE 1:**
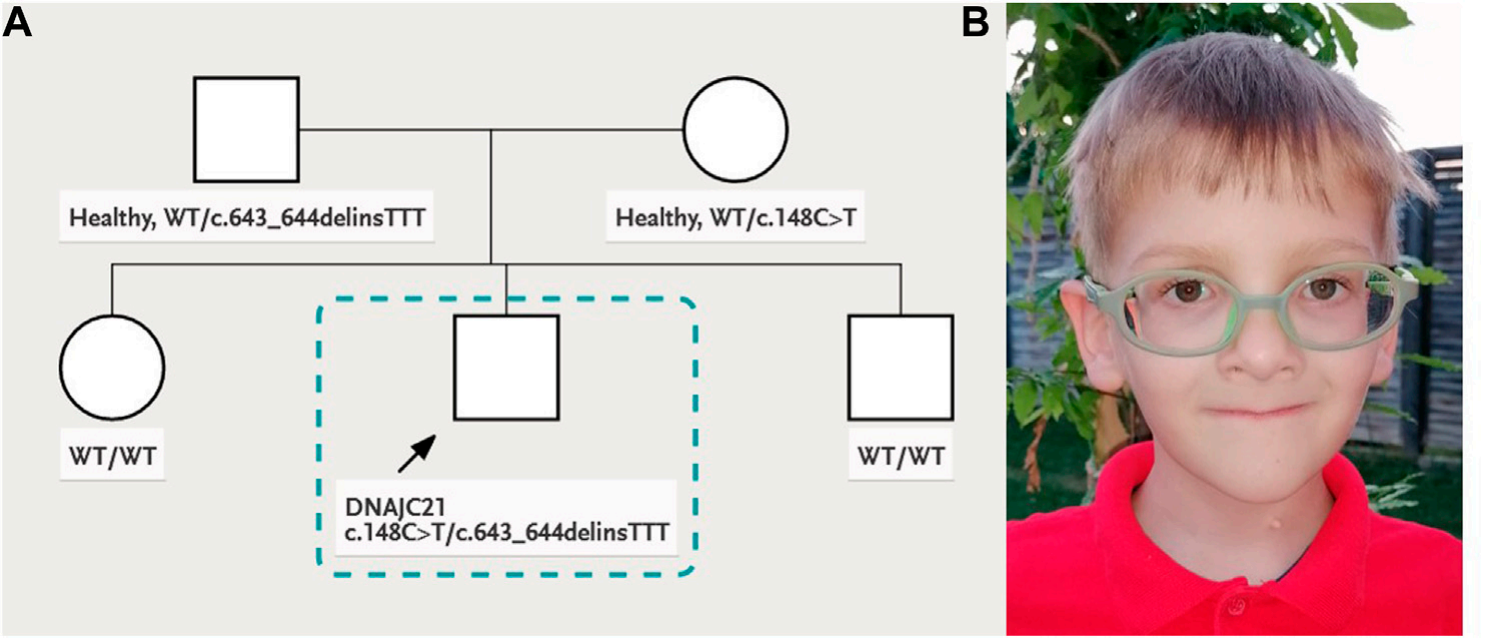
**(A)** Family pedigree and **(B)** patient’s facial phenotype: deep-set eyes, palpebral ptosis, triangular face, and thin lips.

Personal history: The child was born from healthy, young, non-consanguineous parents. The patient’s family history was unremarkable. At 38 weeks of gestation, he was born with a birth weight of 3,000 g, length of 49 cm, head circumference of 36 cm, and APGAR score of 9. The family pedigree is shown in Figure 1A.

### Clinical Evaluation

At 8 y of age, the patient had a particular phenotype with dysmorphic facial features (Figure 1B), triangular face, mild parietal bossing, deep-set eyes, and bilateral asymmetric ptosis. He had optical correction for high myopia. He had thin, sparse, and light-colored hair. His skin was hypohidrotic, dry and rough with lateral–cervical branchial arch defect and skin pedicle. His mouth was small, lips were thin, and lingual frenulum was short, while his dental abnormalities included oligodontia, wide-spaced and cone-shaped teeth, with abnormal enamel. He had slender body habitus with almost absent subcutaneous adipose tissue. His skeletal abnormalities included short stature, pectus carinatum, chest asymmetry, bilateral coxa vara, and genu valgum deformity. He showed muscle hypotonia, normal deep tendon reflexes, and joint hyperlaxity, yet he was able to walk without support, run, and climb stairs. He had normal cognitive capabilities for age. The clinical presentation of the patient in comparison to other 17 people reported in the literature with biallelic *DNAJC21* variants is presented in Table 1.

**TABLE 1:** Clinical and molecular details for 17 reported patients with biallelic *DNAJC21* pathogenic variants, in compassion to our patient. Information was extracted from Tummala et al., 2016; Dhanraj et al., 2017; Bluteau et al., 2018; D’Amours et al., 2018; Ji et al., 2019; Barhoom et al., 2021.

Feature	Characteristics	Reported patient	Number of people with feature/out of total reported
Genomic variant in *DNAJC21* NM_001012339.3	c.94C>G; p:Pro32Ala (homozygous)	-	1/18
	c.100A>G; p:Lys34Glu (homozygous)	-	7/18
	c.148C>T; p.Gln50*/c.643_644delinsTTT; p.Lys215Phefs*71	+	1/18
	c.438-?_894+?del; p:Val148Lysfs*30 (homozygous)	-	1/18
	c.517C>T; p: Arg173* (homozygous)	-	1/18
	c.520C>T; p:Gln174* (homozygous)	-	1/18
	c.544C>T; p.Arg182∗ (homozygous)	-	1/18
	c.793G>T; p:Glu265* (homozygous)	-	1/18
	c.983+1G>T; p:Gly299Alafs*2 (homozygous)	-	2/18
	c.14A>G; p.Tyr5Cys/c.1143_1146del; p:Lys381fs	-	1/18
	Variant not reported by Barhoom et al. (2021)	-	1/18
Gender	Female/male	Male	9/18 were female
Age	Age at first evaluation	8 months	Range 0–12 years
	Age at last follow-up	8 years	Range 14 months to 14 years
Growth	Growth restriction	+	16/18
Hematology and immunologic status	Pancytopenia	+	18/18
	Recurrent infections	+	8/12
	Bone marrow abnormality/hypocellular	+	9/10
	Received bone marrow transplant	-	5/14
Malignancy	Acute myeloid leukemia	-	2/18
Neurological features	Intellectual disability	-	9/18
	Motor delay/hypotonia	+	8/13
	Central nervous system abnormality	-	3/5
	Hearing impairment	-	3/5
Dysmorphic feature	Facial dysmorphism	+	8/10
	Microcephaly	+	6/12
Skeletal abnormalities	Joint hypermobility	+	6/8
	Congenital hip dysplasia	-	6/8
	Osteopenia	-	7/9
Skin and hair abnormalities	Eczema	+	10/10
	Thin sparse hair	+	4/9
Teeth anomalies	Enamel hypoplasia, abnormal tooth shape	+	8/10
Digestive system anomalies	Liver dysfunction	-	5/10
	Pancreatic dysfunction	+	6/11
Genitourinary anomalies	Cryptorchidism	+	3/5
	Kidney anomaly	-	2/6
Eye anomalies	Retinal dystrophy	+	6/8
	Myopia	+	3/5
Other	Short telomeres	+	7/12
	Growth hormone deficiency	+	3/8
	Reported deceased	-	3/18

His personal history immediately after birth showed neonatal hypotonia, poor sucking reflex, and weak and high-pitched cry. In infancy, he had delayed motor milestones. He started to walk around the age of 2 y. The boy had normal intellectual development and attended a normal kindergarten and school. Cerebral ultrasound after birth showed hypoxic ischemic encephalopathy and lateral ventricular asymmetry (left > right). Brain MRI at 2 y of age was normal. EEG evaluation did not show significant variations. Failure to thrive was observed in the first 3 months of life. In infancy and childhood, he had short stature, between −3 SD and −2 SD until the age of 4 y (World Health Organization growth standards). Growth hormone deficit was established at the age of 4 y, based on low IGF-I and positive GH provocation test using insulin. Height improved to −1 SD at the age of 8 y (WHO growth standards) after growth hormone therapy from age 4 y to age 7 y. He also had low weight for height with a body mass index (BMI) that ranged from −3.5 SD to −1.5 SD. Growth charts are presented in the supplementary file. The head circumference was also below −2 SD, proportionate with his short stature. He presented severe localized and general eczema from the age of 5 months, which was attributed to seborrheic dermatitis, atopy, diaper rash, or cow’s milk protein allergy, and received specific treatment. He had cow milk protein restriction for 1 y and 6 months starting from the age of 8 months. During childhood, he had preserved gastrocolic reflex. In general, he had loose stools and sometimes constipation.

The history of illness included repeated infection episodes of urinary tract infection (at 2 weeks and 3 months of age), which was treated with antibiotics. Beginning with the age of 5 months, he had repeated episodes of respiratory tract infections. Some episodes of lower respiratory tract infections were extremely severe and required hospital admissions (eight severe episodes until age 4 y). After initiation of each treatment with antibiotics, he presented diarrhea and weight loss. At birth, the platelet count was 145 × 10^3^/μl. Pancytopenia was first diagnosed at the age of 8 months due to a lower respiratory tract infection episode. At that time, the lowest platelet count was 25 × 10^3^/μl, the lowest leukocyte count was 1,400/mm^3^, the lowest neutrophil count was 240/mm^3^, while the lowest hemoglobin level was 6 g/dl. He had a good response after intensive treatment with erythrocyte and thrombocyte transfusion, antibiotics, corticoids, and immunoglobulins. At the age of 1 y and 3 months, he had another respiratory infection and pancytopenia. However, thrombocytopenia was non-responsive to treatment with corticoids and immunoglobulins. Bone marrow examination performed at age 1 y and 4 months revealed defective megakaryopoiesis (absence of platelet production in most of the megakaryocytes) and frequent mature and reactive lymphocytes. At the age of 1 y and 7 months, he had sepsis with methicillin sensitive *Staphylococcus aureus*, and he presented severe pancytopenia: thrombocytopenia (lowest 9,000/mm^3^), leukocytopenia (lowest 3,180/mm^3^), neutropenia (lowest 1,070/mm^3^), lymphopenia (lowest 1,170/mm^3^), anemia, and reticulocytosis of 71.3%. He recovered after 40 days of inhospital care with intensive treatment (including blood cell transfusions, antibiotics, corticosteroids, immunoglobulins, and other supportive treatments). Immunoglobulin levels were slightly decreased, while B lymphocytes were severely decreased (determined using flow cytometry). After the age of 4 y, the infections were less severe, did not require hospitalization, and did not associate severe pancytopenia; nonetheless, mildly decreased platelet and the neutrophil count were constantly observed.

**FIGURE 2:**
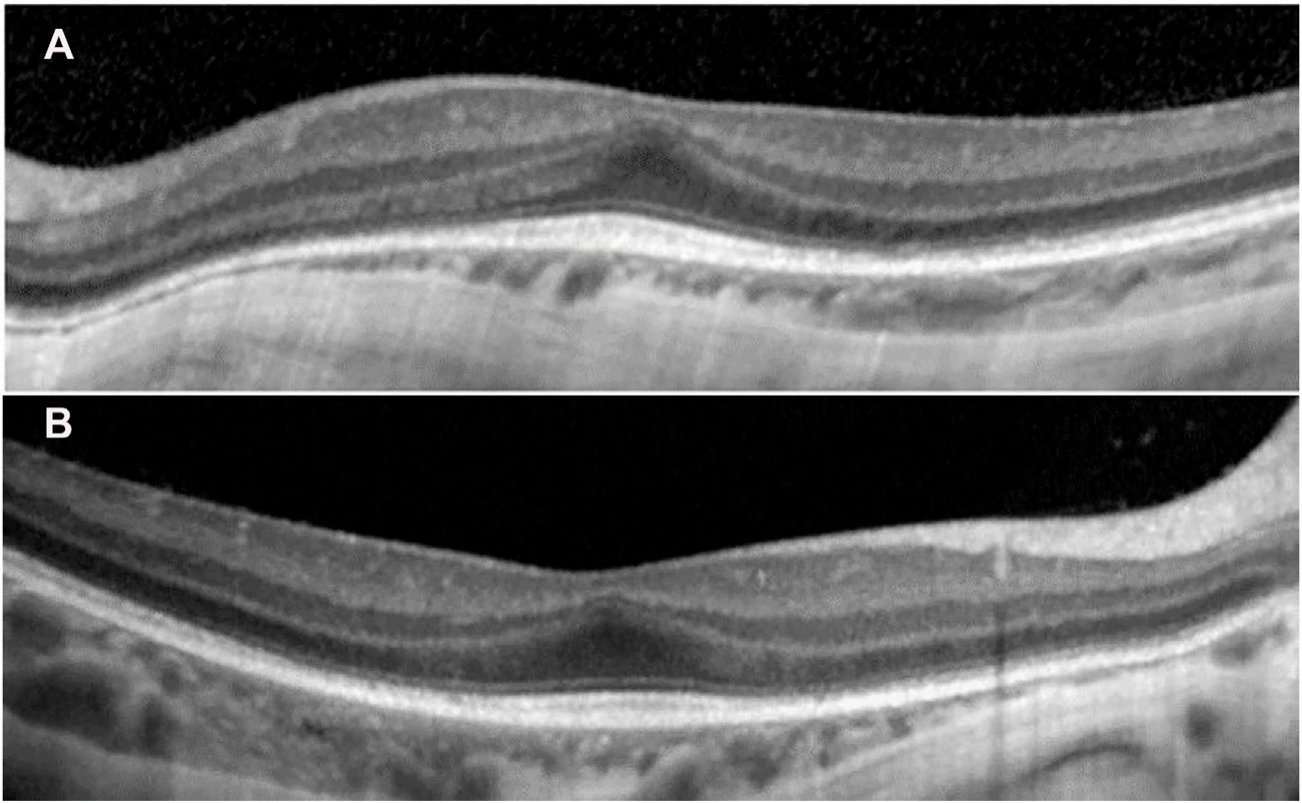
SD-OCT images from horizontal cross section through the retina showed in both eyes **(A)** left eye and **(B)** right eye: increased central foveal thickness (CFT was 251 μm for the left eye and 250 μm for the right eye), absence of the foveal depression, preservation of the inner retinal layers in the fovea (foveal hypoplasia), intact ellipsoid zone (EZ) in the fovea, disruption areas in the external limiting membrane (ELM), decreased outer nuclear layer (ONL) thickness, and weak delimitation of outer segments of photoreceptors and choroidal thinning at the nasal extremity in the retinal pigment epithelium (RPE).

Ophthalmologic evaluation at the age of 4 y led to the diagnosis palpebral ptosis, descending palpebral fissures, high myopia, astigmatism, retinal dystrophy, and pigment deposits in the retina. At age 8 y, he had similar ophthalmologic findings, however with advanced myopia. The best corrected visual acuity at distance using the logMAR VA chart was 0.5 logMAR for the right eye (RE) and 0.3 logMAR for the left eye (LE). For each cycloplegic refraction, a conversion to the spherical equivalent was made (−11.5 diopters for the RE, −12.75 diopters for the LE). His axial length (measured with a Topcon Aladdin Optical Biometer) was 27.39 mm in the RE and 27.33 mm in the LE, respectively. Average axial length for age and gender was 23.31+/−0.48 mm (Tideman et al., 2018). The diopters and increased axial length demonstrated the diagnosis of high myopia. Indirect ophthalmoscopy (with dilated pupil) showed peripapillary chorioretinal atrophy, increased visibility of the choroidal vasculature, optic disc with oval appearance, and macular and peripheral retinal pigment epithelial changes. The color vision was normal (Ishihara’s color plates). Electroretinography (ERG) examinations were performed according to the International Society for Clinical Electrophysiology of Vision standards (after 20 min of adaptation to darkness and 10 min of adaptation to light) using skin electrodes. Full-field ERG recorded a decreased functionality of the rods and the cones in the entire retina of both eyes, confirming the diagnosis of rod-cone dystrophy in both eyes. The visual-evoked potential test revealed increased P wave latency, showing damage to the myelin sheath of both optic nerves. Spectral domain optical coherence tomography was performed with pupils previously dilated (Spectralis OCT, Heidelberg Engineering, Germany). A single-line scan protocol was used for a horizontal cross section through the fovea (ART 100 frames). SD-OCT images from horizontal cross section through the retina showed foveal hypoplasia, conservation of inner retinal layers, and alterations of the external layers and choroidal thinning at the nasal extremity (Figure 2).

## Diagnostic Assessment, Therapeutic Intervention, Follow-Up, and Outcomes

Celiac disease screening (anti-transglutaminase antibodies and anti-endomysial antibodies) was negative. The sweat test for cystic fibrosis could not be performed due to hypohidrosis. Digestive stool analysis repeatedly showed starch in stool after the age of 1 y. Fecal pancreatic elastase at age 4 and 8 y was normal. Amylase, lipase, insulin (in relation to glucose), and glycated hemoglobin were in the normal range. IGF-1 was persistently reduced: at age 4 y, it was 18.40 ng/ml, while at age 8 y, it was 33.1 ng/ml (normal 40–255 ng/ml). Bone age evaluated by hand X-ray was correspondent to his chronological age of 8 y. Thyroid-stimulating hormone and thyroid hormones were normal in all evaluations. Creatine phosphokinase was in a normal range. Transferrin isoelectric focusing, carnitine, and acylcarnitine were normal. Inborn errors of metabolism screening showed plasma amino acids with slightly elevated levels of arginine and asparagine and low ornithine level, while urinary amino acids were normal. The urinary organic acid test showed a slight increase in glutaric acid and 4-OH phenylacetic acid. Lactate was intermittently increased during infections. Calcium metabolism, intact parathyroid hormone, and acid phosphatase were normal.

**FIGURE 3:**
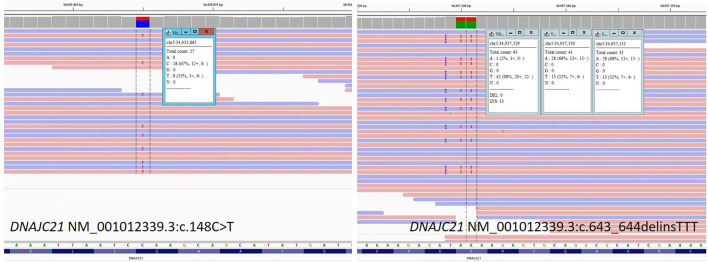
Sequence image for the *DNAJC21* gene: NM_001012339.3:c.148C>T and NM_001012339.3:c.643_644delinsTTT from patient’s whole-genome sequencing.

The genetic laboratory tests performed included: cytogenetic analysis with classic karyotype (46,XY); fluorescent *in situ* hybridization (FISH) for chromosomes 7, 8, and 22 (normal); array CGH (normal male hybridization model); diepoxybutane (DEB) test for Fanconi anemia (negative for chromosomal breakage); Invitae^®^ Sequence analysis and deletion/duplication testing in a panel of 207 genes (2019) for immunodeficiencies (negative); and exome analysis performed in 2016 (negative). Solo whole-genome sequencing performed on DNA extracted from peripheral blood of the patient, using the Illumina platform (Novogene^®^), in 2020, identified two novel, pathogenic variants, in the *DNAJC21* gene: NM_001012339.3:c.148C>T (stopgain) and c.643_644delinsTTT (frameshift) (Figure 3). The two variants were confirmed using the NGS panel (Illumina platform) in the patient’s DNA extracted from blood, in a CLIA-certified laboratory (Invitae^®^). Parental analysis performed at Invitae^®^ demonstrated that the c.148C>T (p.Gln50*) variant was detected in the heterozygous status in the mother, and the c.643_644delinsTTT (p.Lys215Phefs*71) variant was detected in the heterozygous status in the father. Thus, the two null variants were situated on opposite chromosomes in the patient, confirming the compound heterozygous status. Details regarding the whole-genome sequencing method and pathogenicity classification of variants were provided in the supplementary file. NGS testing for the two siblings of the patient did not show any of the variants in *DNAJC21*.

Telomere length was performed on DNA extracted from peripheral blood, using an Absolute Human Telomere Length Quantification qPCR Assay Kit from Sciencell Research Laboratories, for the patient at age 8 y and a matching age and gender healthy control. The analyses were performed in triplicate, following the protocol recommended by the manufacturer. The average telomere length of genomic DNA sample was 0.622 ± 0.088 Mb/diploid cell in the patient, vs. 0.827 ± 0.126 Mb/diploid cell in the control sample (Student’s *t-* test *p* = 0.007).

## Discussion

Bone marrow failure syndromes represent a diverse group of hematological disorders implicating single-line cytopenia or pancytopenia that may lead to the indication for hematopoietic stem cell transplantation (Bluteau et al., 2018). They may be acquired or inherited. Recognizing the inherited nature of bone marrow failure is crucial to prevent inappropriate treatment with immunosuppressant agents and to offer hematopoietic stem cell transplant with an adapted regimen, when needed. Importantly, identifying the genetic cause will help select the healthy donor and provide genetic counseling to the family (Barhoom et al., 2021). Even more, understanding the genetic cause may improve the management of several associated syndromic features. This patient had a late molecular diagnosis that led to some unnecessary treatments, such as cow milk protein restriction for 1 y and 6 months. In addition, the family had another child where informed genetic counseling was not possible in the absence of a molecular diagnosis. Fortunately, none of the siblings carry the variants.

The *DNAJC21* gene was first associated with bone marrow syndrome type 3 in the year 2016 (Tummala et al., 2016). This is one of the reasons why despite several genetic tests performed, a diagnosis was not established for this child. Panels performed did not include the *DNAJC21* gene, while the exome analysis did not identify the two variants in *DNAJC21* to be associated with bone marrow failure. Genome sequencing performed in 2020 aimed to evaluate variants that could have been missed by the exome sequencing; however, it revealed the two exonic variants in the *DNAJC21* gene, at the time recognized to cause disease, thus showing the importance of automated reinterpretation of negative exomes. Even more, there are probably other unknown genes that cause bone marrow failure, as suggested by other authors (Tummala et al., 2016; Dhanraj et al., 2017).

The molecular variation in the *DNAJC21* gene is insufficiently understood. At the time of this report in ClinVar (ClinVar, 2022), a limited number of variants were reported as follows: 28 pathogenic variants (10 single-nucleotide variants (SNV) and 18 copy number variants (CNV)), two likely pathogenic CNV, and 28 variants of unknown significance (24 SNV and 4 CNV). Herein, we report two new pathogenic variants. Better understanding of molecular variation may help the future genotype–phenotype correlation.

Compared to the other people reported in the literature, particular characteristics of this patient were two new variants in the *DNAJC21* gene associated with normal intellect, inability to diagnose pancreatic insufficiency, and a moderate and progressively improved pancytopenia without hematopoietic stem cell transplantation. However, other patients were reported to show spontaneous improvement of cytopenia without bone marrow transplant (D’Amours et al., 2018). The indication and optimal planning for bone marrow transplant remain to be established. An attenuated induction regimen was suggested, considering the probable high sensitivity to chemotherapeutic agents (D’Amours et al., 2018; Barhoom et al., 2021).

Growth failure is a widespread characteristic in people with biallelic variants in the *DNAJC21* gene. The patient’s progressive failure to thrive was observed in the first 3 months of life and was attributed to hypotonia and difficulties in feeding and later due to repeated infections, aggressive treatments, diarrhea, malabsorption, and anemia. Pancreatic insufficiency is difficult to diagnose, however cannot be fully excluded in this patient. Growth hormone therapy treatment may be beneficial when needed; however, considering the disease may predispose to cancer, this therapy might need careful consideration. Long-term follow-up is important to properly understand the risk for malignancy, in people with and without bone marrow transplantation.

A possible retinal phenotype (retinal dystrophy, myopia, and astigmatism) was reported in relation to *DNAJC21* in several other people (Tummala et al., 2016; Dhanraj et al., 2017; D’Amours et al., 2018). This report provides a detailed eye phenotype, showing high myopia, rod–cone retinal dystrophy, altered myelination in the optic nerves, and changes in the retinal layers with foveal hypoplasia, conservation of inner retinal layers, alterations of the external layers, and choroidal thinning at the nasal extremity.

Genome sequencing directed a precise diagnosis that improved patient management and allowed family genetic counseling. This report widens the molecular and clinical understanding of bone marrow failure syndrome type 3, by showing two new variants in the *DNAJC21* gene and a detailed eye phenotype together with a summary of available phenotypic description.

## Caregiver Perspective From the Patient’s Mother

Ever since I first held this wonderful baby, my maternal instinct told me that something was wrong because he did not eat and slept a lot. A urinary tract infection was shortly diagnosed, yet I believed that with treatment, everything would be fine. I never thought he might have a rare disease. This suffering was just the beginning. Severe respiratory infections needing intensive care unit treatment, sepsis, thrombocytopenia, severe myopia, suspicions of different terrible diagnoses, investigations in Romania and Italy, surgery to lower the left testicle, dental extractions, many sedations, and punctures followed. We, as parents, felt continuous fear of losing him. We were stressed, had panic attacks at the slightest symptom of illness, and felt frustrated that we did not know the diagnosis and could not treat him. In 2016, at age 5 y, he was diagnosed with hypodrotic ectodermal dysplasia, based on clinical symptoms. I thought that we finally have a diagnosis and we will know how to help him; however, after 7 y of investigations, the whole-genome sequencing confirmed a different diagnosis for my son: Bone marrow failure type 3. It was surprising to learn that our son is so special and that very few people in the world were reported with this illness. Following this discovery, I feared that my other two children might have the disease. Fortunately, the family screening showed they were not carriers, so we hope that if he will need a bone marrow transplant at some point in his life, one of his siblings can be a donor, if compatible. I would like to thank the entire team of doctors for not giving up in finding a diagnosis and a cure for my son. I am thankful that he now has a diagnosis and a follow-up protocol to be performed by an experienced multidisciplinary team. As parents, we will do everything possible to improve his quality of life. He is a great blessing to our family. He is full of energy and likes to bring joy to people around him. He will be the winner of this terrible diagnosis.

## What is known?

1) Bone marrow failure represents an umbrella diagnosis for several life-threatening disorders. In many people, the etiology remains unknown, leading to numerous tests performed and sometimes inappropriate treatment.
2) In 2016, the *DNAJC21* gene was recognized to cause bone marrow failure syndrome type 3, having phenotypic overlap with Fanconi anemia, dyskeratosis congenita, Shwachman–Diamond syndrome, and Diamond–Blackfan anemia.
3) The phenotype and clinical evolution is insufficiently understood, as only 17 people with biallelic *DNAJC21* gene variants were reported.

## What this report adds?

1) This report widens the molecular and clinical understanding of bone marrow failure syndrome type 3, by showing two new variants in the *DNAJC21* gene and a detailed eye phenotype, together with a comparison with available phenotypic description.

## Data Availability

The datasets for this article are not publicly available due to concerns regarding participant/patient anonymity. Requests to access the datasets should be directed to the corresponding author.
