# Erratum: Novel Biallelic Variants in *DNAJC21* Causing an Inherited Bone Marrow Failure Spectrum Phenotype: An Odyssey to Diagnosis

**DOI:** 10.3389/fgene.2022.930132

**Published:** 2022-05-25

**Authors:** Frontiers Production Office

**Affiliations:** Frontiers Media SA, Lausanne, Switzerland

**Keywords:** DNAJC21 gene, ribosomopathy, bone marrow failure syndrome, Shwachman–Diamond syndrome, telomeres

Due to a production error, there was an error in author **Affiliations** 2 and 6.

A correction has been added to **Acknowledgements**, “The Regional Center of Medical Genetics Timis, Clinical Emergency Hospital for Children “Louis Turcanu” Timisoara, Timis, Romania is part of European Reference Network ERN ITHACA. The Department of Ophthalmology, Municipal Clinical Emergency Hospital of Timisoara, Timisoara, Romania is part of European Reference Network ERN-EYE.”

The publisher apologizes for this mistake. The original version of this article has been updated.

