# Supplementary material for: Case Report: Novel Biallelic Variants in DNAJC21 Causing an Inherited Bone Marrow Failure Spectrum Phenotype: An Odyssey to Diagnosis
Source: Front Genet. 2022 Apr 8;13:870233. doi: 10.3389/fgene.2022.870233 (PMC9023866; doi:10.3389/fgene.2022.870233)
Supplement: Supplementary file 1 [file DataSheet1.docx]

Supplementary file

**Case report: Novel biallelic variants in *DNAJC21* causing an inherited Bone Marrow Failure spectrum phenotype-an odyssey to diagnosis**

Adela Chirita-Emandi^1,2^, Carmen-Angela-Maria Petrescu^3,4^, Cristian G. Zimbru^1,5^, Florina Stoica^6^, Catalin Marian^7,8^, Andreea Ciubotaru^9^, Mihaela Bataneant^3,4^, Maria Puiu^1,2^

1. **Growth parameters:** circles on the graph represent measurements of the patient compared to World Health Organization (WHO) reference.


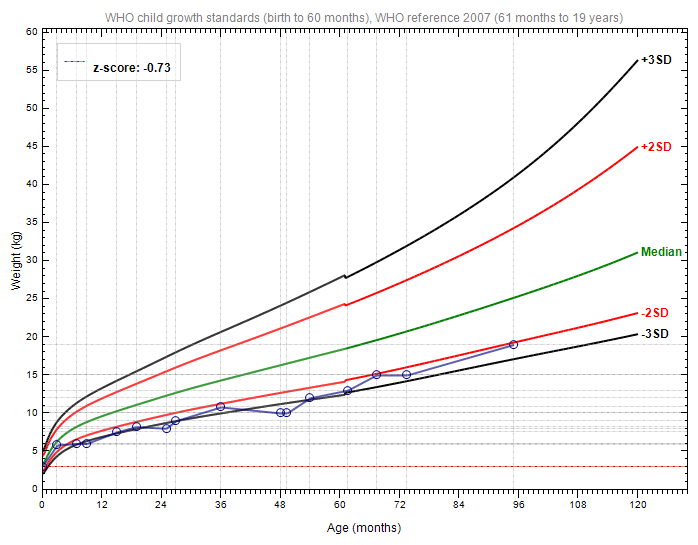


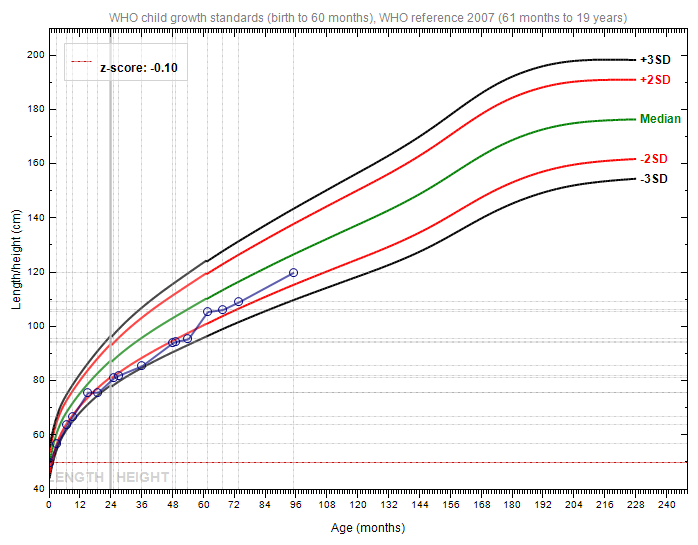


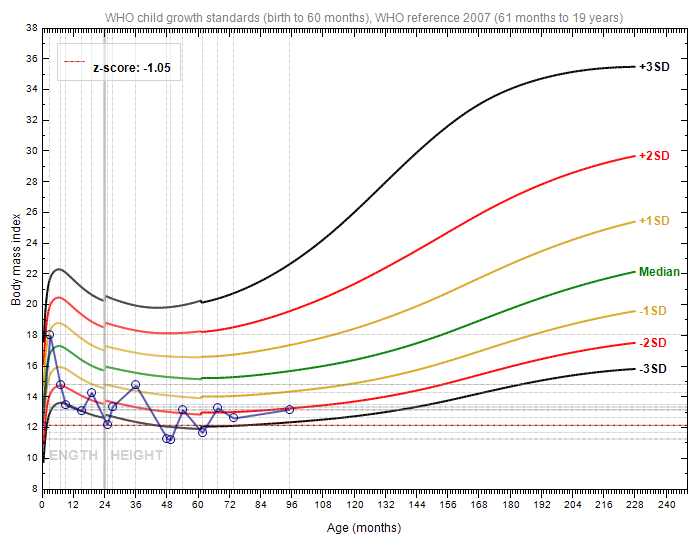


1. Whole genome sequencing method

Whole genome sequencing was carried out in research project, using a next generation sequencing Illumina platform (Novogene, United Kingdom), which allowed genomic DNA analysis. In the first stage, the enzymatic fragmentation of the genomic DNA was performed, followed by amplification and generation of libraries. The coverage level for the target nucleotides in the analyzed sequences was 30X on average. The bioinformatics analysis and the clinical interpretation were performed in the Center of Genomic Medicine Timisoara (University of Medicine and Pharmacy “Victor Babeș” Timisoara), research laboratory. End-to-end bioinformatics algorithms have been implemented, including the alignment of nitrogenous bases, the primary filtering of low-quality readings and probable artifacts, and the annotation of variants to the GRCh38 reference genome. The DRAGEN Illumina platform was used for alignment and identification of structural variants. All variants with a minor allelic frequency (MAF) of less than 1% (gnomAD) and disease-causing variants reported in HGMD®, and ClinVar were considered. The assessment focuses on exon regions and regions flanking exons +/- 20 intronic bases. The entire genome was interrogated for candidate variants with a plausible association to the phenotype. The data analysis was performed at current knowledge, using: UCSC Genome Browser, OMIM (Online Mendelian Inheritance in Man), DGV (Database of Genomic Variants), Clinvar, Varsome, Moon.diploid and others. All transmission patterns were considered, taking into account family history and clinical information. Only variants related to the phenotype for which the patient was referred were reported. Structural variants of unknown significance are not reported. Changes in the number of children (CNV) were also assessed in relation to the patient's phenotype. Variants were interpreted according to the ACMG guideline.

**Variant description for classification *DNAJC21*** MIM 617048 GRCh38:

**NC_000005.9:g.34933970C>T**

**NM_001012339.3:c.148C>T**

**NP_001012339.2:p.(Gln50Ter)**

**Exon 2**

**PVS2 Very Strong:** Null variant (nonsense), in gene *DNAJC21* for which loss-of-function is a known mechanism of disease (gene has 9 pathogenic LOF variants and gnomAD Loss-of-Function Observed/Expected = 0.48 is less than 0.755), associated with Bone marrow failure syndrome 3.

**PM2 Strong**: Using strength Strong because the position is highly conserved (phyloP100way = 7.35 is greater than 7.2). Variant not found in gnomAD exomes. Variant not found in gnomAD genomes (good gnomAD genomes coverage = 31.1).

**PP3 Supporting**: Pathogenic computational verdict based on 5 pathogenic predictions from BayesDel_addAF, DANN, EIGEN, FATHMM-MKL and MutationTaster vs no benign predictions.

**NC_000005.10:g.34937530_34937531delinsTTT**

**NM_001012339.3:c.643_644delinsTTT**

**NP_001012339.2:p.(Lys215PhefsTer71)**

**Exon 5**

**PVS1 Very Strong**: Null variant (frame-shift), in gene *DNAJC21* for which loss-of-function is a known mechanism of disease (gene has 9 pathogenic LOF variants and gnomAD Loss-of-Function Observed/Expected = 0.48 is less than 0.755), associated with Bone marrow failure syndrome 3.

**PM2 Moderate**: Variant not found in gnomAD exomes. Variant not found in gnomAD genomes (good gnomAD genomes coverage = 31.6).

1. Candidate genomic variants in correlation to the patients phenotype from whole genome sequencing

| Chromo-some | Gene | Transcript | Exonic Function | rs | Zygosity | Class |
| --- | --- | --- | --- | --- | --- | --- |
| chr5 | DNAJC21 | NM_001012339.3:c.148C>T  p.(Gln50Ter) | stopgain | NA | het | pathogenic |
| chr5 | DNAJC21 | NM_001012339.3:c.643_644delinsTTT  p.(Lys215PhefsTer71) | frameshift deletion | NA | het | pathogenic |
| chr14 | BMP4 | NM_130851.3:c.124G>T  p.(Ala42Ser) | Non-synonymous SNV | rs140920120 | het | VUS |
| chr19 | CD209 | NM_021155.4:c.523C>T  p.(Arg175Trp) | Non-synonymous SNV | rs773840556 | het | VUS |
| chr2 | DYNC2LI1 | NM_001193464.2:c.852C>G  p. (His284Gln) | Non-synonymous SNV | rs1361706393 | het | VUS |
| chr16 | FBXL8 | NM_018378.3:c.896A>G  p. (Tyr299Cys) | Non-synonymous SNV | rs777343855 | het | VUS |
| chrX | FLNA | NM_001110556.2:c.2362G>A  p. (Glu788Lys) | Non-synonymous SNV | rs911403388 | hom | VUS |
| chr11 | HYOU1 | NM_001130991.3:c.2638G>A  p. (Ala880Thr) | Non-synonymous SNV | rs147237122 | het | VUS |
| chr1 | IBA57 | NM_001010867.4:c.167G>A  p. (Arg56His) | Non-synonymous SNV | rs1035428169 | het | VUS |
| chr14 | MYH7 | NM_000257.4:c.3830G>C  p. (Arg1277Pro) | Non-synonymous SNV | rs397516195 | het | VUS |
| chr22 | TNFRSF13C | NM_052945.4:c.257_268del  p. (Gly86_Leu89del) | Non-frameshift deletion | rs745586116 | het | VUS |

VUS = variant of unknown significance, SNV= single nucleotide variant, het=heterozygous, hom= homozygous
